# Cross-Cultural Comparison of Nonopioid and Multimodal Analgesic Prescribing in Orthopaedic Trauma

**DOI:** 10.5435/JAAOSGlobal-D-20-00051

**Published:** 2020-05-01

**Authors:** Jason D. Young, Abhiram R. Bhashyam, Robert L. Parisien, Quirine Van der Vliet, Rameez A. Qudsi, Jacky Fils, George S. M. Dyer

**Affiliations:** From the Harvard Medical School (Mr. Young, Dr. Bhashyam, Dr. Dyer); the Harvard Combined Orthopaedic Residency Program (Dr. Bhashyam, Dr. Dyer); Boston, MA; the Department of Orthopaedic Surgery (Dr. Parisien), University of Pennsylvania; Philadelphia, PA; the Department of Trauma Surgery (Dr. Van der Vliet), University Medical Center Utrecht; Utrecht, the Netherlands; the Department of Orthopaedics, Nemours (Dr. Qudsi)/A.I. duPont Hospital for Children; Wilmington, DE; and the Department of Orthopaedic Surgery (Dr. Fils, Dr. Dyer), Brigham and Women's Hospital, Boston, MA.

## Abstract

**Background:**

After musculoskeletal injury, US providers prescribe opioids more frequently and at higher dosages than prescribers in the Netherlands and Haiti; however, the extent of variation in nonopioid analgesic prescribing is unknown. The aim of our study was to evaluate how nonopioid prescribing by orthopaedic residents varies by geographic context.

**Methods:**

Orthopaedic residents in three countries in which residents are the primary prescribers of postoperative analgesia in academic medical centers (Haiti, the Netherlands, and the United States) responded to surveys using vignette-based musculoskeletal trauma case scenarios. The residents chose which medications they would prescribe for postdischarge analgesia. We quantified the likelihood and dose of acetaminophen or a nonsteroidal anti-inflammatory drug prescription. We constructed multivariable regressions with generalized estimating equations to describe differences in nonopiate prescription according to country, the resident's sex and training year, and the injury site and age in the test cases.

**Results:**

Compared with residents from the United States, residents from Haiti were more likely to prescribe nonopioids (odds ratio, 3.22 [confidence interval, 1.94 to 5.34], *P* < 0.0001) and residents from the Netherlands nearly always prescribed nonopioids. Of those cases where one or more opioid was prescribed, providers also prescribed a nonopioid (acetaminophen or nonsteroidal anti-inflammatory drug) in 345/603 (57.2%) of US, 152/152 (100%) of Dutch, and 69/97 (71.1%) of Haitian cases (Fisher exact test *P* value <0.0001). Finally, providers prescribed only nonopioids for pain control in 3/348 (0.86%) of US, 32/184 (17.4%) of Dutch, and 107/176 (60.8%) of Haitian cases (Fisher exact test *P* < 0.0001).

**Conclusions:**

When comparing multimodal analgesic patterns, US prescribers prescribed nonopioid analgesics less frequently than prescribers in two other countries, one low income and one high income, either in isolation or in conjunction with opioids.

Musculoskeletal injury is a major contributor to the global burden of preventable injury.^1,2^ In the United States, opioid prescriptions have historically been a mainstay of pain management for traumatic musculoskeletal injuries, leading to an opioid epidemic with significant public health implications.^3-5^ Consequently, there has been increasing scrutiny on opioid prescribing principles with growing clinical and public policy initiatives, focusing on curtailing opioid narcotic prescriptions.^5^ As such, there has been increasing interest in the role of nonopioid medications for postoperative pain management.^6-8^

We are interested in the extent of international variation in analgesic prescribing after orthopaedic surgical treatment for musculoskeletal injuries.^9^ Developing a stronger understanding of how prescribing patterns vary between countries and cultures may clarify the landscape of international prescribing differences and aid in the selection of feasible domestic policy initiatives. For example, we recently demonstrated how opioid prescribing varies significantly between countries, with US providers prescribing opioids more frequently and at higher dosages than prescribers in the Netherlands and Haiti.^10,11^ Yet, that work was limited to opioid prescriptions, and there are few quantitative comparative studies of international postoperative nonopioid prescribing patterns.^7^

Thus, in this study, we examined how nonopioid prescribing varies between countries in total quantity, duration, and coprescription with opioid medications (ie, multimodal analgesia).

## Methods

### Ethics

The Brigham and Women's Hospital institutional review board approved this study.

### Setting

The methodology for this study has previously been published.^11^ In brief, we surveyed orthopaedic residents from nine academic residency programs in three countries (the United States, the Netherlands, and Haiti) between April and November 2017, using a set of 10 musculoskeletal trauma vignettes (SDC 1, http://links.lww.com/JG9/A76). We chose to focus on residents because they are the primary postoperative analgesic prescribers at academic centers and thus enable us to more accurately evaluate prescribing patterns in academic clinical practice.^12,13^ A vignette-based design, compared with standard survey-based studies, has previously been demonstrated to reduce social desirability bias and to provide greater variable manipulation. This study design is hypothesized to provide greater construct validity and reliability.^14^ Of the 10 cases presented, five were of men and five were of women. In addition, five of the cases were of younger-aged patients (<45 years old), whereas five were older-aged patients (>70 years old). Finally, two each of the 10 cases described trauma to different anatomical sites (ankle, femur, wrist, tibial shaft, and tibial plateau). Respondents indicated their initial postoperative discharge pain regimen for each case in the form of administration dose (mg), number of tablets, and duration (days) for each case. Refill requests were not solicited in the cases.

Respondents were asked to indicate what analgesic medications they would prescribe for each case. Their choices were one or more of the following nonopioids (acetaminophen and ibuprofen) and opioids (acetaminophen with codeine #3, tramadol, oxycodone, and hydromorphone). Owing to the differences in medication availability in Haiti, the list was modified in conjunction with a native Haitian physician for Haitian respondents: codeine replaced acetaminophen with codeine #3, diclofenac replaced ibuprofen, and Paracetamol replaced Tylenol.

The vignettes were translated into French and Dutch, and then back-translated by native speakers for quality control. We collected responses via the Qualtrics survey platform in the United States and the Netherlands (Qualtrics.com). Pen-and-paper surveys were used to solicit responses in Haiti.

### Participants

Our study population was restricted to surgical residents managing orthopaedic trauma in all countries. Our rationale was that residents are the primary prescribers of postoperative analgesics at academic centers and decide the medications and their dose and duration. Consequently, all residents responding to one or more cases were included in the study. All incomplete and uninterpretable responses were excluded from the study.

### Statistical Analysis

Owing to the correlation between responses to cases from each respondent, we performed regressions with generalized estimation equations with compound symmetry working correlation to characterize differences in nonopioid analgesic prescriptions between countries, respondent demographics (sex and training year), and case characteristics (younger vs older patient and site of injury). Alpha was set to *P* < 0.05 without adjustment for multiple comparisons, given the exploratory nature of the analysis. Sample sizes were dictated by the maximum availability of respondents and case responses. SAS statistical software (SAS Institute) was used for all analyses.

## Results

### Study Sample

Participant characteristics are presented in SDC 2, http://links.lww.com/JG9/A77. A total of 139 residents participated in the study. Eight-five (61%) were from the United States, 30 (22%) were from the Netherlands, and 24 (17%) were from Haiti. Seventy-four (87%) of US, 15 (50%) of Dutch, and 21 (91%) of Haitian respondents were men. Response rates, defined as respondents providing at least one complete case response, were 67% (US respondents), 50% (the Netherlands respondents), and 79% (Haiti respondents). Because of the low-resource setting in Haiti and the concern for underprescription by the residents, Haitian respondents were asked to indicate whether they believed their prescriptions in the cases were adequate for pain control, of which 11 of 15 (73%) residents responded in the affirmative.

Nonopioid prescription among survey respondents is presented in Table 1. In all countries, acetaminophen was the most commonly prescribed nonopioid.

**Table 1:** Descriptive Statistics of Prescriptions by Country

	No. of Completed Cases (%)			Total (n = 995)
	United States (n = 606)	Netherlands (n = 185)	Haiti (n = 204)	
Prescriptions				
Acetaminophen	349 (57.6%)	181 (97.8%)	141 (69.1%)	671 (67.4%)
NSAIDs^a^	55 (9.1%)	91 (49.2%)^b^	71 (34.8%)	217 (21.8%)

NSAIDS, nonsteroidal anti-inflammatory drug

a Ibuprofen (the United States, the Netherlands) or diclofenac (Haiti).

### Nonopioid Analgesic Dosing: Multivariable Analyses

We found significant differences in acetaminophen prescriptions between residents of the three countries. Residents from the Netherlands prescribed more acetaminophen per day (3695 mg, confidence interval [CI], 3414 to 3976) than did residents from the United States (2953 mg [CI, 2619 to 3288]) or Haiti (1899 mg [CI, 1599 to 2199]) (*P* < 0.0001) (Table 2). Similar results were obtained for the amount of total acetaminophen prescribed per case (SDC 3, http://links.lww.com/JG9/A78).

**Table 2:** Least Square Means for the Total Acetaminophen Prescription per Day From the (A) Multivariable Model With GEEs and (B) Final GEE Model

	(A) Multivariable Model With GEEs		(B) Final GEE Model	
	Acetaminophen Estimate/day (95% Confidence Interval)	*P*	Acetaminophen Prescription (mg) Estimate/day (95% Confidence Interval)	*P*
Country				
United States	2994 (2557-3432)	<0.0001	2953 (2619-3288)	<0.0001
Netherlands	3925 (3623-4227)		3695 (3414-3976)	
Haiti	1891 (1344-2438)		1899 (1599-2199)	
Sex		0.72		
Female	3006 (2316-3697)			
Male	2867 (2688-3047)			
Training year		0.95		
1	3031 (2405-3658)			
2	2911 (2463-3359)			
3	2982 (2541-3423)			
4	3039 (2380-3698)			
≥5	2722 (2061-3382)			
Age		0.27		
<40 yr	2893 (2564-3222)			
>70 yr	2981 (2627-3335)			
Injury site		0.52		
Ankle	2892 (2551-3234)			
Femur	2949 (2594-3304)			
Wrist	2906 (2559-3254)			
Tibial shaft	3064 (2690-3439)			
Tibial plateau	2872 (2525-3220)			

GEE = generalized estimation equation

When comparing nonsteroidal anti-inflammatory drug (NSAID) prescriptions between the different countries, no differences were observed in ibuprofen prescribed per day or total ibuprofen prescription per case (SDC 4, http://links.lww.com/JG9/A79) between the United States and the Netherlands. Interaction effects were not significant for all variables in our generalized estimation equation models.

### Nonopioid Analgesic Likelihood of Prescription: Odds Ratios

Odds ratios (ORs) were constructed through multilevel logistic regressions with clustering to examine the likelihood of prescribing nonopioid medications (Table 3). Compared with residents from the United States, residents from Haiti were more likely to prescribe nonopioids (OR 3.22 [CI, 1.94 to 5.34], *P* < 0.0001). ORs could not be constructed for Dutch prescribers because of the insufficient number of cases in which nonopioids were not prescribed. In a combined analysis of all three countries, female prescribers were found to be more likely to prescribe nonopioid analgesic medications than their male counterparts (OR: 1.86 [CI, 1.04 to 3.32], *P* = 0.036). In addition, compared with first-year residents, senior residents (years 2 to 4) were found to be more likely to prescribe nonopioids (all ORs >2, all *P* < 0.005); however, fifth-year residents were found to be less likely to prescribe nonopioid analgesics (OR: 0.32 [CI, 0.19 to 0.55], *P* < 0.0001).

**Table 3:** Odds Ratios of Likelihood of Prescribing Nonopioids

	OR (95% CI)	*P*
Country		
Netherlands	NA^a^	NA^a^
Haiti	3.22 (1.94-5.34)	<0.0001
United States	Reference	Reference
Sex		
Female	1.86 (1.04-3.32)	0.036
Male	Reference	Reference
Training year		
2	2.16 (1.31-3.59)	0.0027
3	3.33 (1.99-5.55)	<0.0001
4	2.38 (1.37-4.11)	0.0019
5	0.32 (0.19-0.55)	<0.0001
1	Reference	Reference
Age		
<40	1.08 (0.78-1.5)	0.65
>70	Reference	Reference
Injury site		
Femur	1 (0.6-1.67)	0.99
Wrist	1.07 (0.64-1.79)	0.81
Tibial shaft	1.05 (0.63-1.76)	0.84
Tibial plateau	1 (0.6-1.68)	0.99
Ankle	Reference	Reference

CI = confidence interval; OR = odds ratio

a NA = Not Applicable because nearly all (184/185) residents from the Netherlands prescribed nonopioids for the vignette cases.

Similar results were obtained when examining only NSAID prescription likelihood (diclofenac or ibuprofen) (Table 4), except Dutch prescribers, compared with those from the United States, were also found to be much more likely to prescribe NSAIDs (OR 29.96 [CI, 14.94 to 60.07], *P* < 0.0001). In addition, younger patients were more likely to be prescribed NSAIDs than older patients (OR 3.04 [CI, 1.98 to 4.67], *P* < 0.0001). When examining the likelihood of acetaminophen prescription (Table 4), similar results were obtained based on the resident training year, whereas we found no significant differences between countries, prescriber sex, or patient age.

**Table 4:** Odds Ratios of the Likelihood of Prescribing (A) NSAIDS (Ibuprofen or Diclofenac) or (B) Acetaminophen

	(A) Likelihood of Prescribing NSAIDs		(B) Likelihood of Prescribing Acetaminophen	
	OR (95% CI)	*P*	OR (95% CI)	*P*
Country				
Netherlands	29.96 (14.94-60.07)	<0.0001	NA^a^	NA^a^
Haiti	3.25 (2.03-5.18)	<0.0001	1.12 (0.75-1.68)	0.58
United States	Reference	Reference	Reference	Reference
Sex				
Female	2.91 (1.66-5.11)	0.00019	1.58 (0.93-2.68)	0.092
Male	Reference	Reference	Reference	Reference
Training year				
2	0.35 (0.16-0.77)	0.0087	1.97 (1.2-3.21)	0.0069
3	4.36 (2.51-7.6)	<0.0001	2.26 (1.44-3.56)	0.0004
4	1.89 (0.95-3.76)	0.071	2.16 (1.28-3.64)	0.0038
5	NA	NA	0.28 (0.17-0.47)	<0.0001
1	Reference	Reference	Reference	Reference
Age				
<40	3.04 (1.98-4.67)	<0.0001	0.97 (0.71-1.32)	0.85
>70	Reference	Reference	Reference	Reference
Injury site				
Femur	0.97 (0.51-1.83)	0.92	1.08 (0.66-1.74)	0.77
Wrist	0.89 (0.47-1.69)	0.72	1.22 (0.75-1.98)	0.43
Tibial shaft	0.88 (0.46-1.69)	0.71	1.13 (0.69-1.83)	0.63
Tibial plateau	1.08 (0.57-2.03)	0.82	0.95 (0.59-1.54)	0.84
Ankle	Reference	Reference	Reference	Reference

CI = confidence interval; NSAID = nonsteroidal anti-inflammatory drug; OR = odds ratio

a NA = Not Applicable because nearly all (181/185) residents from the Netherlands prescribed acetaminophen for the vignette cases.

### Multimodal Analgesia

Rates of opioid prescription are presented in SDC 5, http://links.lww.com/JG9/A80. For each country, all cases were stratified by (1) whether an opioid was prescribed and (2) whether any nonopioid was prescribed for analgesia (Table 5). In total, providers prescribed opioids in 603/606 (99.5%) of cases from the United States, 152/185 (82.2%) of cases from the Netherlands, and 97/204 (47.6%) of cases from Haiti (Fisher exact test *P* < 0.0001). Of those cases where one or more opioid was prescribed, providers also prescribed a nonopioid (acetaminophen, diclofenac, or ibuprofen) in 345/603 (57.2%) of the US, 152/152 (100%) of Dutch, and 69/97 (71.1%) of Haitian cases (Fisher exact test *P* value <0.0001). Finally, providers prescribed only nonopioids for pain control in 3/348 (0.86%) of the US, 32/184 (17.4%) of Dutch, and 107/176 (60.8%) of Haitian cases (Fisher exact test *P* < 0.0001).

**Table 5:** Rates of Opioid and Nonopioid Prescribing for Multimodal Analgesia

United States				Netherlands				Haiti			
Prescribed ≥1 Nonopioid Medications	Prescribed ≥1 Opioid Medications			Prescribed ≥1 Nonopioid Medications	Prescribed ≥1 Opioid Medications			Prescribed ≥1 Nonopioid Medications	Prescribed ≥1 Opioid Medications		
	No	Yes	Total		No	Yes	Total		No	Yes	Total
No	0	258	258	No	1	0	1	No	0	28	28
Yes	3	345	348	Yes	32	152	184	Yes	107	69	176
Total	3	603	606	Total	33	152	185	Total	107	97	204

## Discussion

Previous work has demonstrated significant variation in opioid prescribing between countries, with US providers prescribing opioids more commonly and at higher doses.^10,11^ To better contextualize US opioid prescribing, this study sought to examine how nonopioid prescribing varies between countries in total quantity, duration, and coprescription with opioid medications. Our study demonstrates clinically relevant differences in nonopioid and multimodal analgesic prescribing between the United States, Haiti, and the Netherlands. Comparatively, US prescribers are less likely to prescribe acetaminophen and NSAIDs for postoperative analgesia. In addition, US residents prescribing acetaminophen prescribed less per day than Dutch residents. When comparing multimodal analgesic patterns, US prescribers prescribed nonopioids with opioids less frequently and nonopioid analgesics in isolation less frequently than Dutch or Haitian residents.

Acetaminophen is a commonly used, relatively safe analgesic that is opioid-sparing when used for acute pain in combination with other nonopioids. Acetaminophen is an important component of multimodal analgesic prescribing guidelines for surgical orthopaedic trauma.^15^ We found that US residents prescribed acetaminophen for postoperative pain less frequently than did residents from the Netherlands (Table 4). ORs for a comparison of the likelihood of acetaminophen prescribing between US and Dutch residents could not be constructed because of the high proportion of Dutch cases (97.8%) having acetaminophen prescriptions. This latter finding is reflected in the Dutch pain management guidelines because the first-line of medical management of postoperative and nociceptive pain begins with acetaminophen, with escalation to NSAIDs and opioids, respectively.^16^ We found the doses of acetaminophen prescribed by US and Dutch residents fall within the FDA and European guidelines at a maximum of 4 g/day.^16,17^ However, our results indicate that US prescribers are prescribing significantly less than their Dutch counterparts, although Dutch residents are on average prescribing acetaminophen at levels which are at or below the FDA safe dosing limits. Taken together, these findings would suggest that US prescribers may be underprescribing acetaminophen for multimodal analgesia postoperatively.

Haitian residents prescribe significantly lower doses of acetaminophen than their US counterparts. The reason is unclear and may relate to resource constraints, which have been previously noted regarding analgesic medications in the country.^18^ In addition, cultural differences in patient responses and expectations of medical management of postoperative pain have been documented and may also contribute.^19,20^ Future work is needed to clarify the extent of analgesic medication resource limitations in Haiti and their impact on postoperative pain management.

We found that US residents were less likely to prescribe NSAIDs compared with the Dutch and Haitian residents (Table 4). Historically, studies have demonstrated the NSAID impairment of bone healing via COX-2 inhibition, raising clinical concern domestically regarding their role in fracture care.^21,22^ However, most studies supporting this finding have been conducted in animal models, with studies in humans failing to corroborate these concerns.^23-25^ In addition, given the limited length of NSAID prescribing for perioperative and postoperative pain in orthopaedic trauma, avoiding NSAIDs out of concern for impairing bone healing may be overstated because previous studies of short-term NSAID use in the spine literature have demonstrated no effect on union rates.^26^ In the Netherlands, the Dutch national protocol for postoperative pain has interpreted the literature similarly, stating it cannot be concluded that NSAIDs impair bone healing based on the current literature.^27^ Hence, Dutch prescribers widely use NSAIDs in the treatment of postoperative pain after surgical management of fractures. As our findings among US prescribers might suggest, limiting NSAID use may theoretically encourage greater opioid-based analgesia, thereby putting patients at greater risk from complications of opioid use. Certainly, further work will be needed to clarify how differential NSAID prescribing might affect the nonunion rates in orthopaedic trauma. However, given the absence of convincing evidence demonstrating a clinical difference in nonunion rates, particularly between the United States and Europe, our findings would support a liberalization of NSAID prescribing domestically in the management of postoperative pain.

We also found that female prescribers were more likely to prescribe nonopioids, driven by an increased likelihood of NSAID prescribing (Tables 3 and 4). In addition, younger patients were more likely to be prescribed NSAIDs (Table 4). Although the age differences align with current clinical conventions concerning older patients being less likely to tolerate NSAIDs given a higher propensity to sustain adverse effects (GI symptoms, increased bleeding risk, etc.), it is less clear why a difference exists between provider sexes, and this may be a focus of further study.^28,29^

We found that anatomic location did not impact nonopioid prescribing (Table 4). A previous analysis conducted by this group demonstrated differential opioid prescribing for cases of orthopaedic trauma based on anatomic location,^11^ but the reasons why such a differential prescribing pattern does not hold true for nonopioids remain unclear. Previous studies have suggested the possibility of dose efficacy for nonopioids in pain management, although nonopioid-dose response has not been directly studied within an orthopaedic context.^30^ Future work will be needed to better understand the relationship between patient-reported pain and nonopioid dosing for postoperative analgesia.

In this study, we demonstrated significant differences in multimodal analgesic prescribing. Specifically, although US residents prescribed opioids more frequently, they also prescribed nonopioids in combination less frequently, and prescribed nonopioids in isolation less frequently than Dutch or Haitian residents (Table 5). Remarkably, in 100% of cases in which an opioid was prescribed, Dutch residents also prescribed a nonopioid for multimodal analgesia. This prescribing behavior is not only consistent with Dutch analgesic prescribing guidelines, but it also seems to align with recent studies indicating that multimodal analgesia both results in better pain control postoperatively and decreased opioid usage compared with opioid monotherapy.^31,32,33,34,35^ In comparison, US residents used opioid monotherapy in 42.8% of cases. This finding may at least partly explain the findings from our previous study which indicated higher opioid doses and prescription durations prescribed among the US residents for postoperative analgesia.^10,11^ Prescribing opioid monotherapy is not without risks because previous work has noted an increased likelihood of adverse events associated with higher levels of opioid exposure.^36^ In addition, recent work has indicated that opioids may also limit bone healing and callus formation and thus raise concern for opioid use at high doses or for prolonged durations.^37,38^ A corollary to these findings is that the US counterparts differs significantly from its international counterparts in the rate of nonopioid monotherapy for analgesia. Nonopioids alone were prescribed in 17.4% of Dutch and 60.8% of Haitian cases compared with 0.86% of US cases. Although the high rate of nonopioid-only prescriptions in Haiti may be partly because of the resource constraints, a recent randomized controlled trial demonstrated that the use of NSAIDs with acetaminophen may be just as effective as opioids for the management of postoperative pain, in addition to reducing adverse events associated with opioid use.^18,39^ Future comparative work is needed to draw definitive conclusions concerning analgesic recommendations. However, our findings suggest that differences in opioid prescribing between the US and international counterparts may at least partly be explained by the lower rates of multimodal analgesic practices and nonopioid prescribing. Furthermore, this study highlights how prescribers in the United States, compared with other countries, may be underutilizing acetaminophen and NSAIDs both in isolation and combination and that an area of potential improvement may be in improving multimodal analgesic prescribing practices for postoperative pain.

This study has several limitations. Our study does not capture the actual prescribing data or patient-reported pain, thus limiting the extent to which we can translate our findings to clinical practice. Indeed, previous studies have indicated the possible presence of patient-related and culturally-mediated factors influencing patient satisfaction with pain relief,^40^ which may influence the prescriber behavior in ways which our analysis is not able to assess. However, we believe that the strength of this study is our use of standardized case vignettes, which enable a more direct international comparison of prescribing practices than would be feasible via observational data alone. Second, our study is limited to resident prescribing and not that of attendings. However, most opioid medications prescribed by surgeons are prescribed by trainees,^41^ and although we recognize that residents are not permitted to prescribe opioids without direct attending oversight, previous studies have indicated that, in practice, resident prescribing occurs most commonly without direct communication with the attending.^42^ Third, the lack of resources in Haiti poses challenges for the interpretation of our comparative study.^43^ However, most responding Haitian residents (73%) believed that their indicated prescriptions were sufficient to manage postoperative pain. Furthermore, our use of theoretical vignettes may limit the impact of resource constraints on what is and is not prescribed. Fourth, we obtained response rates of 51% to 79% in our surveyed countries, which may subject our study to nonresponse bias. Fifth, we did not assess the use of other nonopioid medications such as gabapentin, pregabalin, or cannabinoids.^44^ Finally, our sample size in Haiti and the Netherlands was much smaller compared with the United States, largely because there are far fewer orthopaedic residents in those countries.

## Conclusion

In conclusion, this study helps contextualize the US analgesic prescribing practices within an international context. We found that US prescribers are less likely to prescribe NSAIDs for postoperative analgesia compared with their international counterparts, are less likely than Dutch prescribers to prescribe acetaminophen, and prescribe a lower dose of acetaminophen compared with Dutch prescribers. In addition, the US prescribers prescribed nonopioid analgesics in isolation less frequently than Dutch or Haitian residents. Finally, when prescribing opioids, the US residents prescribed nonopioids in combination less frequently than did Dutch or Haitian residents. These findings are consistent with our previous work demonstrating an overreliance on opioids for postoperative analgesia in the United States and further demonstrate the need for the standard prescribing guidelines that liberalize nonopioid prescribing patterns while limiting opioid monotherapy.

## Supplementary Material

SUPPLEMENTARY MATERIAL
