# Supplementary material for: Cross-Cultural Comparison of Nonopioid and Multimodal Analgesic Prescribing in Orthopaedic Trauma
Source: J Am Acad Orthop Surg Glob Res Rev. 2020 May 1;4(5):e20.00051. doi: 10.5435/JAAOSGlobal-D-20-00051 (PMC7434039; doi:10.5435/JAAOSGlobal-D-20-00051)

**Supplemental Digital Content 1:** Survey Instrument (images courtesy of http://boneschool.com/)

Assessing Analgesic Prescription Patterns Among Orthopedic Surgery Residents in Haiti and the US

Participant Code: Gender: □ Female □ Male

Year of Residency: Hospital:

**Introduction:** Below you will find a series of mock cases of orthopedic trauma. Every case requires open reduction and internal fixation. Please indicate which analgesic you would prescribe for the patient’s pain if you were treating this patient at discharge or in your usual practice setting(s). In addition, indicate the dose, frequency, and duration of the medication. You may choose more than one medication.

**Note: None of the patients have ever taken opioid medications.**

**Case 1**: An otherwise healthy 26 year old male presents with right ankle pain after a soccer injury. Radiographs reveal a bimalleolar ankle fracture. Open reduction internal fixation is

performed.

|  | Dose: | Frequency: | Duration: |
| --- | --- | --- | --- |
| □ codeine |  |  |  |
| □ diclofenac |  |  |  |
| □ hydromorphone |  |  |  |
| □ oxycodone |  |  |  |
| □ paracetamol (acetaminophen) |  |  |  |
| □ tramadol |  |  |  |
| □ no medication | | | |


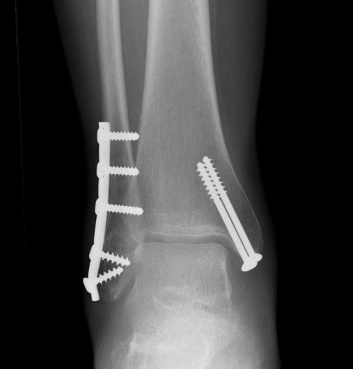


**Case 2**: An otherwise healthy 73 year old female is struck by a bus and is brought to the ED. She is found to have a right midshaft femoral shaft fracture. Within 24 hours, open reduction internal fixation was performed with an intramedullary nail.

|  | Dose: | Frequency: | Duration: |
| --- | --- | --- | --- |
| □ codeine |  |  |  |
| □ diclofenac |  |  |  |
| □ hydromorphone |  |  |  |
| □ oxycodone |  |  |  |
| □ paracetamol (acetaminophen) |  |  |  |
| □ tramadol |  |  |  |
| □ no medication | | | |


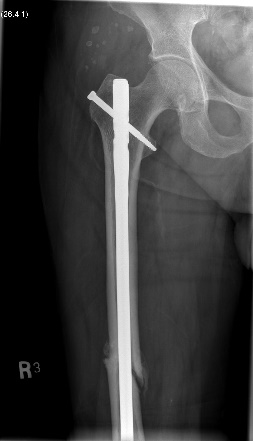


**Case 3:** An otherwise healthy 22 year old female presents with right wrist pain and deformity after an accident at work. Radiographs demonstrated a displaced distal radius fracture. She was treated with open reduction internal fixation using a volar locking plate.

|  | Dose: | Frequency: | Duration: |
| --- | --- | --- | --- |
| □ codeine |  |  |  |
| □ diclofenac |  |  |  |
| □ hydromorphone |  |  |  |
| □ oxycodone |  |  |  |
| □ paracetamol (acetaminophen) |  |  |  |
| □ tramadol |  |  |  |
| □ no medication | | | |


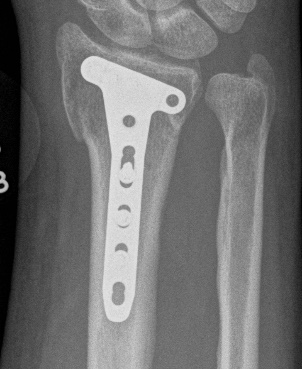


**Case 4:** An otherwise healthy 32 year old male complains of severe right leg pain after a motor vehicle collision. Standard AP and lateral radiographs were taken and the patient was diagnosed with a tibial shaft fracture. Open reduction internal fixation was performed with an intramedullary nail.

|  | Dose: | Frequency: | Duration: |
| --- | --- | --- | --- |
| □ codeine |  |  |  |
| □ diclofenac |  |  |  |
| □ hydromorphone |  |  |  |
| □ oxycodone |  |  |  |
| □ paracetamol (acetaminophen) |  |  |  |
| □ tramadol |  |  |  |
| □ no medication | | | |


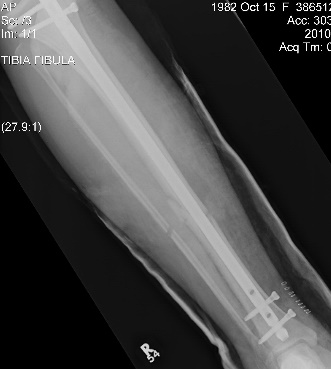


**Case 5**: An otherwise healthy 82 year old male is struck by a car and presents with right knee pain. Standard AP and lateral knee radiographs were taken. The patient is diagnosed with a right tibial plateau fracture. Open reduction internal fixation is performed.

|  | Dose: | Frequency: | Duration: |
| --- | --- | --- | --- |
| □ codeine |  |  |  |
| □ diclofenac |  |  |  |
| □ hydromorphone |  |  |  |
| □ oxycodone |  |  |  |
| □ paracetamol (acetaminophen) |  |  |  |
| □ tramadol |  |  |  |
| □ no medication | | | |


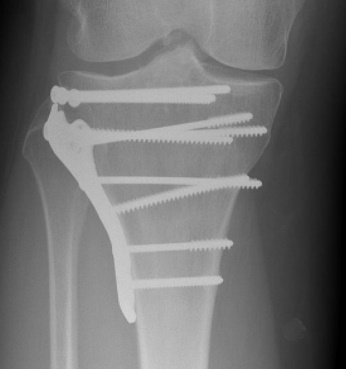


**Case 6**: An otherwise healthy 77 year old male presents with right ankle pain after a fall. A right bimalleolar ankle fracture is found on X-ray. Open reduction internal fixation is performed.

|  | Dose: | Frequency: | Duration: |
| --- | --- | --- | --- |
| □ codeine |  |  |  |
| □ diclofenac |  |  |  |
| □ hydromorphone |  |  |  |
| □ oxycodone |  |  |  |
| □ paracetamol (acetaminophen) |  |  |  |
| □ tramadol |  |  |  |
| □ no medication | | | |


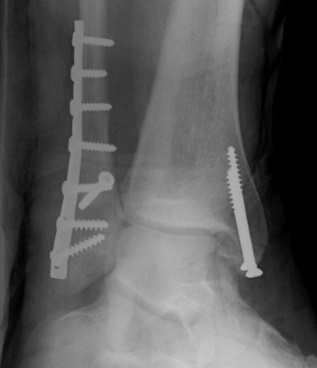


**Case 7**: An otherwise healthy 37 year old female is brought to the ED after a motor vehicle collision. She is found to have a right midshaft femoral shaft fracture. Open reduction internal fixation was performed with an intramedullary nail within 24 hours of presentation.

|  | Dose: | Frequency: | Duration: |
| --- | --- | --- | --- |
| □ codeine |  |  |  |
| □ diclofenac |  |  |  |
| □ hydromorphone |  |  |  |
| □ oxycodone |  |  |  |
| □ paracetamol (acetaminophen) |  |  |  |
| □ tramadol |  |  |  |
| □ no medication | | | |


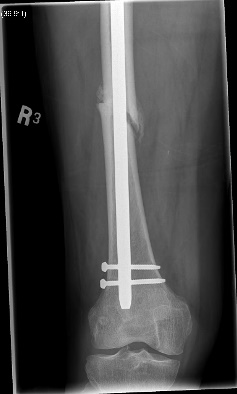


**Case 8:** An otherwise healthy 79 year old female fell on her outstretched hand and presents with right wrist pain and deformity. Radiographs demonstrated a displaced distal radius fracture. She was treated with open reduction internal fixation using a volar locking plate.

|  | Dose: | Frequency: | Duration: |
| --- | --- | --- | --- |
| □ codeine |  |  |  |
| □ diclofenac |  |  |  |
| □ hydromorphone |  |  |  |
| □ oxycodone |  |  |  |
| □ paracetamol (acetaminophen) |  |  |  |
| □ tramadol |  |  |  |
| □ no medication | | | |


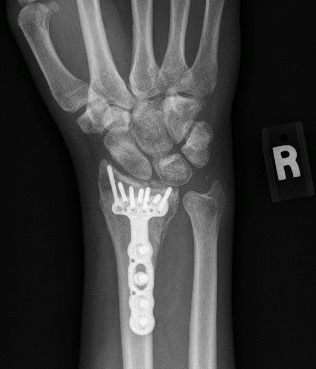


**Case 9:** An otherwise healthy 80 year old male is struck by a motorcycle and complains of severe left leg pain. Standard AP and lateral radiographs reveal a tibial shaft fracture. Open reduction internal fixation was performed with an intramedullary nail.

|  | Dose: | Frequency: | Duration: |
| --- | --- | --- | --- |
| □ codeine |  |  |  |
| □ diclofenac |  |  |  |
| □ hydromorphone |  |  |  |
| □ oxycodone |  |  |  |
| □ paracetamol (acetaminophen) |  |  |  |
| □ tramadol |  |  |  |
| □ no medication | | | |


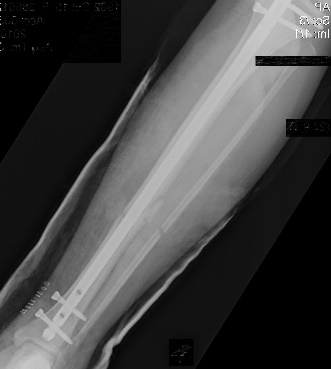


**Case 10**: An otherwise healthy 41 year old male presents with left knee pain after being struck by a motorcycle. Standard AP and lateral knee radiographs were taken and the patient was diagnosed with a left tibial plateau fracture. Open reduction internal fixation was performed.

|  | Dose: | Frequency: | Duration: |
| --- | --- | --- | --- |
| □ codeine |  |  |  |
| □ diclofenac |  |  |  |
| □ hydromorphone |  |  |  |
| □ oxycodone |  |  |  |
| □ paracetamol (acetaminophen) |  |  |  |
| □ tramadol |  |  |  |
| □ no medication | | | |


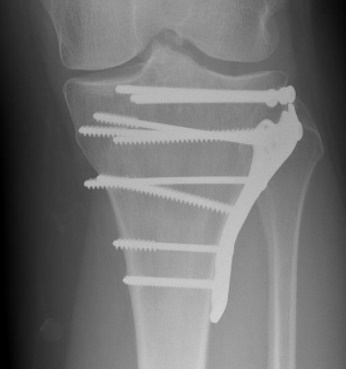

Supplement: SUPPLEMENTARY MATERIAL [file jg9-4-e20.00051-s001.docx]
