# Supplementary material for: Cross-Cultural Comparison of Nonopioid and Multimodal Analgesic Prescribing in Orthopaedic Trauma
Source: J Am Acad Orthop Surg Glob Res Rev. 2020 May 1;4(5):e20.00051. doi: 10.5435/JAAOSGlobal-D-20-00051 (PMC7434039; doi:10.5435/JAAOSGlobal-D-20-00051)
Supplement: SUPPLEMENTARY MATERIAL [file jg9-4-e20.00051-s002.docx]

**Supplemental Digital Content 2:** Descriptive Statistics of Survey Respondents

|  | No. (%) | | |  |
| --- | --- | --- | --- | --- |
|  | U.S.  (n=85) | Netherlands  (n=30) | Haiti  (n=24) | Total  (n=139) |
| Sex |  |  |  |  |
| Male | 74 (87.1%) | 15 (50.0%) | 21 (87.5%) | 110 (79.1%) |
| Female | 11 (12.9%) | 15 (50.0%) | 2 (8.3%) | 28 (20.1%) |
| Missing | 0 | 0 | 1 (4.2%) | 1 (0.7%) |
| Full response to ≥1 cases |  |  |  |  |
| Yes | 58 (68.2%) | 15 (50.0%) | 20 (83.3%) | 93 (66.9%) |
| No | 27 (31.8%) | 15 (50.0%) | 4 (16.7%) | 46 (33.1%) |
| Training year |  |  |  |  |
| 1 | 15 (17.6%) | 4 (13.3%) | 6 (25.0%) | 25 (18.0%) |
| 2 | 19 (22.4%) | 6 (20.0%) | 1 (4.2%) | 26 (18.7%) |
| 3 | 19 (22.4%) | 1 (3.3%) | 10 (41.7%) | 30 (21.6%) |
| 4 | 11 (12.9%) | 1 (3.3%) | 3 (12.5%) | 15 (10.8%) |
| 5 | 21 (24.7%) | 0 | 0 | 21 (15.1%) |
| Missing | 0 | 18 (60.0%) | 4 (16.7%) | 22 (15.8%) |

*Chi-squared test p-value.
