# Supplementary material for: Cross-Cultural Comparison of Nonopioid and Multimodal Analgesic Prescribing in Orthopaedic Trauma
Source: J Am Acad Orthop Surg Glob Res Rev. 2020 May 1;4(5):e20.00051. doi: 10.5435/JAAOSGlobal-D-20-00051 (PMC7434039; doi:10.5435/JAAOSGlobal-D-20-00051)
Supplement: SUPPLEMENTARY MATERIAL [file jg9-4-e20.00051-s003.docx]

**Supplemental Digital Content 3**: Least Square Means for Total Acetaminophen Prescription from the (A) Multivariable Model with GEEs, and (B) Final GEE Model

|  | Multivariable Model with GEEs | | Final GEE Model | | |
| --- | --- | --- | --- | --- | --- |
|  | Acetaminophen Estimate (95% Confidence Interval) | P Value | Acetaminophen Prescription Estimate (mg) (95% Confidence Interval) | | P Value |
| **Country** |  |  |  |  | |
| **U.S.** | **44852 (34954, 54750)** | **0.0006** | **46166 (35739, 56594)** | **0.0002** | |
| **Netherlands** | **51964 (38002, 65926)** |  | **49902 (41989, 57815)** |  | |
| **Haiti** | **18958 (10393, 27523)** |  | **18242 (11020, 25464)** |  | |
| Sex |  | 0.86 |  |  | |
| Female | 37985 (25595, 50374) |  |  |  | |
| Male | 39198 (33809, 44586) |  |  |  | |
| Training year |  | 0.37 |  |  | |
| 1 | 42185 (28546, 55825) |  |  |  | |
| 2 | 51351 (32015, 70687) |  |  |  | |
| 3 | 32579 (24250, 40908) |  |  |  | |
| 4 | 37571 (27328, 47815) |  |  |  | |
| ≥5 | 29269 (15067, 43472) |  |  |  | |
| Age |  | 0.30 |  |  | |
| <40 yr | 37790 (31235, 44346) |  |  |  | |
| >70 yr | 39392 (32635, 46150) |  |  |  | |
| Injury site |  | 0.09 |  |  | |
| Ankle | 35173 (28292, 42053) |  |  |  | |
| Femur | 38263 (31478, 45048) |  |  |  | |
| Wrist | 35974 (29102, 42846) |  |  |  | |
| Tibial shaft | 44070 (35360, 52780) |  |  |  | |
| Tibial plateau | 39476 (32817, 46136) |  |  |  | |
