# Supplementary material for: Cross-Cultural Comparison of Nonopioid and Multimodal Analgesic Prescribing in Orthopaedic Trauma
Source: J Am Acad Orthop Surg Glob Res Rev. 2020 May 1;4(5):e20.00051. doi: 10.5435/JAAOSGlobal-D-20-00051 (PMC7434039; doi:10.5435/JAAOSGlobal-D-20-00051)
Supplement: SUPPLEMENTARY MATERIAL [file jg9-4-e20.00051-s004.docx]

**Supplemental Digital Content 4:** Least Square Means for Total Ibuprofen Prescription and Total Ibuprofen Prescription per Day per Case from the Multivariable Model with GEEs

|  | Ibuprofen Estimate (95% Confidence Interval) | P Value | Ibuprofen Estimate/day (95% Confidence Interval) | P Value |
| --- | --- | --- | --- | --- |
| Country |  |  |  |  |
| U.S. | 31453 (4152, 58754) | 0.27 | 3890 (-1225, 9005) | 0.40 |
| Netherlands | 5463 (-7776, 18703) |  | 614 (-1710, 2938) |  |
| Sex |  | 0.20 |  | 0.32 |
| Female | 4880 (-17671, 27432) |  | 146 (-4052, 4345) |  |
| Male | 32036 (8898, 55174) |  | 4358 (-620, 9336) |  |
| Training year |  | 0.63 |  | 0.83 |
| 1 | 11569 (-11436, 34574) |  | 1313 (-2170, 4797) |  |
| 2 | 20700 (4350, 37050) |  | 1204 (-2056, 4463) |  |
| 3 | 33746 (-4627, 72120) |  | 5758 (-2329, 13846) |  |
| 4 | 7817 (-14098, 29732) |  | 732 (-3342, 4806) |  |
| Age |  | 0.34 |  | 0.67 |
| <40 yr | 18896 (6782, 31010) |  | 2277 (109, 4445) |  |
| >70 yr | 18020 (5758, 30282) |  | 2227 (-5, 4458) |  |
| Injury site |  | 0.19 |  | 0.64 |
| Ankle | 19487 (6239, 32735) |  | 2521 (-21, 5063) |  |
| Femur | 23255 (6407, 40104) |  | 3006 (-252, 6264) |  |
| Wrist | 9096 (-6984, 25176) |  | 438 (-2620, 3496) |  |
| Tibial shaft | 20560 (6286, 34834) |  | 2651 (-34, 5337) |  |
| Tibial plateau | 19892 (4815, 34970) |  | 2643 (-110, 5397) |  |
