# Supplementary material for: Cross-Cultural Comparison of Nonopioid and Multimodal Analgesic Prescribing in Orthopaedic Trauma
Source: J Am Acad Orthop Surg Glob Res Rev. 2020 May 1;4(5):e20.00051. doi: 10.5435/JAAOSGlobal-D-20-00051 (PMC7434039; doi:10.5435/JAAOSGlobal-D-20-00051)
Supplement: SUPPLEMENTARY MATERIAL [file jg9-4-e20.00051-s005.docx]

**Supplemental Digital Content 5**. Number of cases with prescribed opioid medications among three countries.

| Country | Cases where ≥ 1 opioid medications were prescribed | | | | All |
| --- | --- | --- | --- | --- | --- |
|  | No | | Yes | |  |
|  | N | % | N | % | N |
| US | 3 | 0.50 | 603 | 99.50 | 606 |
| Netherlands | 33 | 17.84 | 152 | 82.16 | 185 |
| Haiti | 107 | 52.45 | 97 | 47.55 | 204 |
| All | 143 | 14.37 | 852 | 85.63 | 995 |
